# Overexpression of dihydroflavonol 4-reductase (*CoDFR*) boosts flavonoid production involved in the anthracnose resistance

**DOI:** 10.3389/fpls.2022.1038467

**Published:** 2022-11-09

**Authors:** Chaochen Yang, Pengfei Wu, Yongqing Cao, Bingbing Yang, Linxiu Liu, Juanjuan Chen, Renying Zhuo, Xiaohua Yao

**Affiliations:** The Research Institute of Subtropical of Forestry, Chinese Academy of Forestry, Hangzhou, China

**Keywords:** *Camellia oleifera*, dihydroflavonol 4-reductase (*CoDFR*), anthracnose, flavonoid, disease resistance

## Abstract

The outbreak of anthracnose caused by *Colletotrichum* spp. represents a devastating epidemic that severely affects oil tea (*Camellia oleifera*) production in China. However, the unknown resistance mechanism to anthracnose in *C. oleifera* has impeded the progress of breeding disease-resistant varieties. In this study, we investigated the physiological responses of resistant and susceptible lines during *C. gloeosporioides* infection. Our results showed that the accumulation of malondialdehyde (MDA), catalase (CAT), superoxide dismutase (SOD), and peroxidase (POD) in both disease-resistant and susceptible lines increased by *C. gloeosporioides* infection. Also, disease-resistant lines exhibited lower MDA, but higher POD, SOD, and CAT activities compared to susceptible lines. The accumulation of flavonoids in both resistant and susceptible *C. oleifera* leaves increased following *C. gloeosporioides* infection, and the increase was greater in resistant lines. Further, we identified and functionally characterized the dihydroflavonol 4-reductase (*CoDFR*) from the resistant *C. oleifera* line. We showed that the full-length coding sequence (CDS) of *CoDFR* is 1044 bp encoding 347 amino acids. The overexpression of *CoDFR* in tobacco altered the expression of flavonoid biosynthetic genes, resulting in an increased flavonoid content in leaves. *CoDFR* transgenic tobacco plants exhibited increased anthracnose resistance. Furthermore, the transgenic plants had higher salicylic acid content. These findings offer potential insights into the pivotal role of *CoDFR* involved in flavonoid-mediated defense mechanisms during anthracnose invasion in resistant *C. oleifera*.

## Introduction

*Colletotrichum* spp. was first discovered by Penzig in 1882, with common distribution mainly in tropical and subtropical areas (Weir et al., 2012). As a plant pathogen, it can cause a variety of woody and herbaceous plant diseases (Cannon et al., 2007; Liu et al., 2022a). *Colletotrichum* spp. mainly causes anthracnose. However, several reports suggest that it can cause other diseases, such as strawberry *(Fragaria × ananassa)* and banana (*Musa paradisiaca* L.) fruit rot, cowpea *(Vigna unguiculata)* brown spot, etc. (Bankole and Adebanjo, 1996; Ureña-Padilla et al., 2002; Jat et al., 2013). *Colletotrichum* spp. has a wide range of hosts and can quickly spread under warm and humid conditions. Meanwhile, the immune mechanism of the host is not well understood, which poses a challenge to control anthracnose (Chen et al., 2022; Yang et al., 2022). Recently, anthracnose has become a worldwide threat, which seriously restricts crop quality and yield improvement, resulting in huge economic losses (Jiang et al., 2021). In the 1960s, Chinese scholars carried out preliminary studies on anthracnose disease in *Camellia oleifera*, majorly focusing on the exploration of pathogen sources, transmission routes, prevention, and control methods (Yuan et al., 1963).

*Camellia oleifera* Abel (*C. oleifera*) is an evergreen broad-leaved shrub belonging to the genus *Camellia* (Theaceae) (Lin et al., 2022a). It is mainly distributed in the tropical and subtropical areas of southern China and has a long history of cultivation and consumption (Zhang et al., 2008; Qu et al., 2020). As an important woody oil tree species, *C. oleifera* has developed rapidly in China (Yang et al., 2017; Lin et al., 2022b). However, with an increase in oil tea tree cultivation areas, there are prevailing reports on the occurrence of anthracnose as the main disease in oil tea production areas. The annual output of oil tea tree seeds has been reduced by 20%-40% due to anthracnose, and even 80% in severe cases, causing significant losses to local foresters and oil tea tree enterprises (Zhang et al., 2019; Li and Li, 2020; Chen et al., 2022). Many approaches have been used to reduce the losses caused by anthracnose, including agronomic measures and host resistance, but these measures are not always feasible (Xu et al., 2020; Chen et al., 2022). Chemical pesticides can prevent anthracnose, but resistance of the pathogens develops easily and may be harmful to human health (Tian et al., 2019; Chen et al., 2022). Mining disease-resistant genes to cultivate resistant varieties is the most economical and effective measure to prevent and control anthracnose.

Imbalanced reactive oxygen species (ROS) production is a result of multifaceted responses when plants are exposed to biotic or abiotic stress. Excess ROS can result in lipid peroxidation in plant cells, which leads to the accumulation of membrane lipid peroxidation byproduct, malondialdehyde (MDA). MDA is an important index of the antioxidant capacity in plants. Plants develop resistance mechanisms to eliminate ROS and avoid oxidative damage. For example, catalase (CAT), peroxidase (POD), superoxide dismutase (SOD), and other enzymatic activities in plant cells are also induced in response to stress. In addition, ROS components can act as signaling substances to induce the production of disease-resistant metabolites (Feng et al., 2008; Zhang et al., 2016; Watkins et al., 2017). Flavonoid biosynthesis is one of the most important metabolic processes in plants, which plays a key role in the interaction between plants and pathogens (Mansfeld et al., 2017; Dai et al., 2019; Li et al., 2021). The biosynthesis of flavonoids begins with phenylalanine, which is catalyzed by phenylalanine-related core biosynthetic enzymes to synthesize major flavonoids and their derivatives (Saito et al., 2013). Dihydroflavonol 4-reductase (DFR) is a key enzyme in the flavonoid pathway for the synthesis of anthocyanins, catechins, and procyanidins (Tian et al., 2017). It controls the flux of each branch pathway for the production of these three substances (Punyasiri et al., 2004; Jiang et al., 2020; Ruan et al., 2022). The *DFR* genes have been cloned and identified in most plants, for example, *Ipomoea batatas Lam.* (Liu et al., 2017), *Euphorbia pulcherrima* (Gu et al., 2018) and *Brassica napus L.* (Kim et al., 2017). The functions of *DFR* genes in these plants has been shown to be associated with flavonoid accumulation. Furthermore, previous studies indicated that overexpression of *DFR* increased the tolerance of transgenic plants to biotic and abiotic stresses (Kumar et al., 2013; Kim et al., 2017). In previous study we found that *C. gloeosporioides* infection caused changes in the expression profiles of *CoDFR* in disease-resistant *C. oleifera* lines. However, the role of *CoDFR* in resistance to anthracnose remains unclear.

In this study, we evaluated the physiological responses and flavonoid content in resistant and susceptible lines of *C. oleifera* after *C. gloeosporioides* infection. Further, we cloned *CoDFR*, which was differentially expressed in a previous transcriptome screen of the resistant *C. oleifera* line during *C. gloeosporioides* inoculation (PRJNA775660, Yang et al., 2022). The expression profiles of *CoDFR* in different tissues at different time points were studied after *C. gloeosporioides* infection. In addition, the ectopic expression of *CoDFR* in tobacco promoted flavonoid production, thereby increasing plant resistance to anthracnose.

## Materials and methods

### Plant and fungal materials and treatment conditions

Two-year-old cutting seedlings of ‘CL150’ (resistant line) and ‘CL102’ (susceptible line) of *C. oleifera* were grown in the Climate Chamber of Research Institute of Subtropical Forestry, Chinese Academy of Forestry, (CAF, N30°05’, E119°96’), Hangzhou, China. The cuttings, planted into plastic basins (14 cm in diameter × 11 cm in height) containing peat moss (Klasmann, Germany), were incubated under the following conditions; 26°C temperature, 90% relative humidity, and 16/8 hours of light/dark cycle. The pathogenic *C. gloeosporioides* strain was donated by the research group of forest protection at Central South University of Forestry and Technology (Li et al., 2017). Leaves from both lines were inoculated, as described by Yang et al. (2022). Briefly, the new leaves of *C. oleifera* were sterilized with 75% alcohol and rinsed with sterile water. Then, two wounds were punctured with sterilized large-headed needles on both sides of the leaf veins, and 10 µL of sterile 1.0% glucose solution was injected through the wounds. Finally, the wound was covered with fungal hyphae cake (5 mm) cultured for 5-7 days. Each treatment was done on six seedlings per line and repeated three times.

### Leaf antioxidant enzyme activity

The whole *C. oleifera* leaves were collected at 0 hour-post inoculation (hpi), 24 hpi, 48 hpi, 72 hpi, 96 hpi, and 120 hpi. 2.0 g of the samples were weighed and stored in the centrifuge tube (placed immediately in liquid nitrogen and then stored in a refrigerator at -80°C) to measure the enzyme activity. Malondialdehyde (MDA) content and peroxidase (CAT), superoxide dismutase (SOD), and peroxidase (POD) activities were determined using the corresponding reagent kits (Jiancheng, Nanjing, China. http://www.njjcbio.com/) (Li et al., 2013). There were four treatment groups (CL150CK, CL150T, CL102CK and CL102T), with three biological replicates at each time point for each treatment group. Data are presented as mean ± standard deviation (SD) of three biological replications.

### Flavonoid content estimation

The *C. oleifera* leaves were collected at 72 hpi, then dried at 60°C to a constant weight in all the samples and used to determine the total phenol (TP) and total flavonoid (TF) contents. The contents of total phenolic (TP) and total flavonoid (TF) were estimated following the manufacturer’s instructions (Jiancheng, Nanjing, China. http://www.njjcbio.com/). Briefly, weigh 0.2 g of the sample after passing through 40 mesh sieve, add 20.0 mL of extraction solution (60% ethanol absolute), shake at 60 °C for 2 h, 10,000 g, centrifuge at 25 °C for 10 min, and measure the absorbance value of the extract at 502 nm by spectrophotometer (UV-3200, Mapada, China) for the calculation of TF content. Weigh 0.1 g of the sample, add 2.0 mL of the extraction solution, and use a spectrophotometer to determine the absorbance value of the extraction solution at 760 nm for calculating the TP content. There were four treatment groups (CL150CK, CL150T, CL102CK and CL102T), each with three biological replicates. Data are presented as mean ± SD of three biological replications.

### Bioinformatic analysis

The amino acid sequences of DFRs from different plant species were obtained from the NCBI (https://www.ncbi.nlm.nih.gov/) database. Multiple sequence alignment of DFR protein sequences was performed using TBtools software (Chen et al., 2020). The MAGE X was used to construct the evolutionary tree using the following parameters: maximum likelihood method, Poisson correction, and bootstrap value 1,000 (Tamura et al., 2013).

### Quantitative real-time PCR (qRT-PCR) analysis

Gene-specific primers were designed using Primier Express 3.0.1 and are listed in **Supplementary Table S1**. About 0.8 µg of total RNA was reverse transcribed into cDNA using the PrimeScript 1st strand cDNA synthesis kit (Takara, Japan). The qRT-PCR reactions were carried out using the PrimeScript RT reagent qPCR kit (TaKaRa, Japan) on the Quant Studio 7 FlexReal-Time PCR System (Applied Biosystems, USA). Relative quantification was evaluated using the 2^-(ΔΔCT)^ method (Zhou et al., 2013). Each experiment was repeated three times.

### Subcellular localization and overexpression analysis

The full-length *CoDFR* coding sequence was amplified from *C. oleifera* cDNA, and the CDS region was cloned without a stop codon. The target gene was finally cloned into the pCambia1300-GFP vector by homologous recombination to constitute a fusion expression vector. The CaMV35S::CoDFR recombinant plasmids were inserted into *Agrobacterium tumefaciens* strain GV3101, which were then co-cultivated with tobacco leaf sections as previously described (Klee et al., 1987). Genomic PCR using primers specific for both the Hygromycin gene and the *CoDFR* gene was performed to verify positive transformants. pCambia1300 empty vector was used as the control, and subcellular localization experiments were performed as described previously (Wang et al., 2022).

### Analysis of plant resistance to the pathogen

Three transgenic tobacco lines expressing a significantly high level of CoDFR were screened and wild-type was used as a control to evaluate plant resistance to *C. fructicola*. About six-week-old tobacco grown in the climate chamber of the Institute of Subtropical Forestry, CAF, was used for the inoculation experiment. The tobaccos, planted into plastic basins (11.5 cm in diameter × 11 cm in height) containing peat moss (Klasmann, Germany), were maintained at 26 °C and a relative humidity of 90% with cycles of 16 h light and 8 h darkness. Depending on leaf size, 6-10 spots were infested per leaf and 3-4 leaves were infested per tobacco plant. There were four lines (WT, D4, D11 and D12), and each line has at least 24 inoculation spots. Data are presented as mean ± SD. The spot diameters at 96 hpi were measured using the crossover method, and then the leaves were collected for measuring the flavonoid content. There were four lines (WT, D4, D11 and D12), and each line was sampled three times independently. The flavonoid content data are presented as mean ± SD of three biological replications.

### UPLC–MS/MS analysis

Over 500 mg of tobacco leaves infected with *C. fructicola* were collected at 96 hpi for targeted metabolome determination. The Luming biological technology co., LTD (Shanghai, China) provided targeted metabolomics services. The specific method is as follows. The lyophilized sample was accurately weighed 50 mg and placed in a 2.0 mL centrifuge tube. The sample was ground (60 HZ) for 2 min. To each sample tube, about 600 μL mixture of water/methanol (1/2, v/v) was added, followed by the addition of 400 μL chloroform. Ultrasonic extraction was performed in an ice water bath for 20 min. After centrifugation for 10 min (4°C, 15620 g), 300 μL of the supernatant was collected and loaded into EP tubes. Then, a 400 μL mixture of water/methanol (1/2, v/v) was added to the residue, and samples were placed at -20°C for 2 min. Centrifugation was repeated once for a final collection of 600 μL of extracts. 300 μL of the supernatant in a brown glass vial was dried using a freeze concentration centrifugal dryer. 300 μL mixture of methanol and water (7/18, vol/vol) was added to each sample, containing L-2-chlorophenylalanine as an internal standard. The samples were vortexed for 30 s and ultrasonicated at ambient temperature for 2 min followed by centrifugation at 15620 g, 4 °C for 5 min. The supernatants (200 μL) from each tube were collected using crystal syringes, filtered through 0.22 μm microfilters, and transferred to LC vials.

UPLC-ESI-MS/MS (ultra-performance liquid chromatography-electrospray tandem mass spectrometry) analysis method was used for qualitative and quantitative detection of flavonoid phenolic metabolites. The specific analysis conditions and methods are mentioned below. Chromatographic conditions: the chromatographic system was an AB ultra-performance liquid chromatograph with a Waters UPLC HSS T3(100×2.1 mm, 1.8 μm) liquid chromatographic column based on phenolic properties, with an injection volume of 5 μL. Mobile phase A (0.1% formic acid in aqueous solution), mobile phase B (acetonitrile). Metabolite quantification was analyzed using the multiple reaction detection (SRM) mode in triple quadrupole mass spectrometry. The default parameters in SCIEX OS-MQ software (SCIEX) were used for automatic identification and integration of each MRM transition, which was followed by manual inspection. The concentration of each substance was calculated by the one-point external standard method (Labadie et al., 2020). For the two treatment groups (overexpression and wild type line, three biological replicates per treatment group), T-test (Student’s t test) and Fold change analysis were used to compare the differential metabolites between the two groups. Metabolites with P<0.05 and |log2(FC)|≥1.0 were considered as differentially accumulated metabolites. Data are presented as mean ± SD of three biological replications.

### Statistical analysis

Microsoft Office Excel 2016 was used to process the data. GraphPad Prism 9.0 was used to create the plots, and SPSS 22.0 software was used to test for significant differences. The Shapiro-Wilk test was used to check whether the data met the normality for ANOVA. If the data met the assumptions, then we further conduct ANOVA. If the data don’t meet the assumptions, Kruskal-Wallis test was used. Duncan *post hoc* test was performed for pairwise comparisons of means at a 0.05 significance level.

## Results

### Changes in physiological characteristics of *C. oleifera* leaves after *C. gloeosporioides* infection

**Figure 1:**
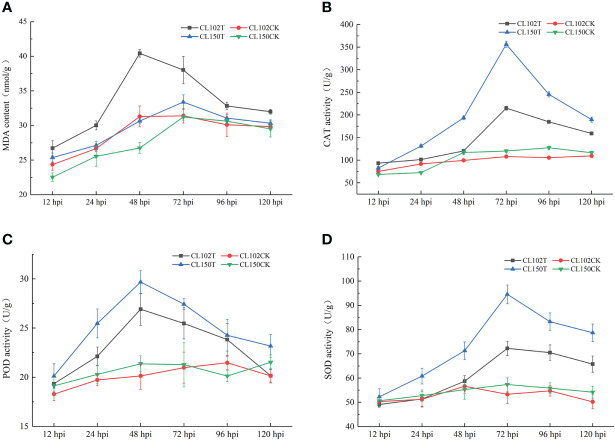
Measurement of the physiological indicators of different timepoints. **(A)** The content of MDA. **(B)** The activities of CAT. **(C)** The activities of POD. **(D)** The activities of SOD. Each value represents mean ± standard error (n = 3).

To explore the physiological response in *C. oleifera* leaves during *C. gloeosporioides* infection, we measured the MDA content and determined the CAT, POD, and SOD activities in different samples collected at 12, 24, 48, 72, 96, and 120 hpi. After *C. gloeosporioides* infection, the MDA content in both CL150 (resistant line) and CL102 (susceptible line) showed an increasing trend. The maximum MDA level was observed in CL102 at 48 hpi, whereas in CL150, it reached the highest at 72 hpi, 40.42 nmol•g-1, and 33.39 nmol•g-1, respectively. It was increased in both the lines after infection, as compared to the control plants (uninoculated CL150 named CL150CK and uninoculated CL102 named CL102CK). Overall, the MDA content in CL102T (inoculated CL102) was significantly higher than that in CL150T (inoculated CL150) (P< 0.05) (**Table S2**; **Figure 1A**). The CAT activity in oil tea resistant and susceptible lines increased. Among these, the CL102T reached 215.01 U•g -1 at 72 hpi, significantly higher than the CL102CK. The CL150T reached 356.22 U•g -1 at 72 hpi, significantly higher than the CL150CK (**Table S2**). The CAT activity in CL150T was found to be higher than that in CL102T, CL150CK, and CL102CK and varied a little during the whole experiment (**Figure 1B**). At 12 hpi, POD activity in CL102T and CL150T was significantly higher than CL102CK and CL150CK (**Table S2**; **Figure 1C**). At 48 hpi, the POD activity in CL150T was the maximum (29.67 U•g -1), which was significantly higher than that of CL102T (26.91 U•g -1). The maximum values of POD activity were obtained at 48 hpi in CL150T and CL102T, which decreased after 48 hpi. The POD activity was elevated in CL150T than that in CL102T at all the time points (**Figure 1C**). At 72 hpi, the activity of SOD reached a peak value of 94.53 U•g -1 in the leaves of CL150T, which was significantly higher than that in CL150CK. The SOD activity in CL102T reached a peak value of 72.25 U•g -1 at 72 hpi, and the SOD activity in the leaves of CL150T was 1.34 times that of CL102T. The SOD activity in CL150T was significantly higher than the CL102T (**Table S2**; **Figure 1D**).

### Flavonoid content estimation in disease-resistant and susceptible lines of *C. oleifera*

To determine the accumulation of flavonoids in resistant and susceptible lines, we measured the flavonoid content in *C. oleifera* leaves at 3dpi. The flavonoid content in CL150T was 54.51 mg/g, while in CL102T was 47.54 mg/g. The contents of procyanidin B1, procyanidin B2, and procyanidin B3 in CL150T were 204.49, 54.68, and 107.22 μg/g, while in CL102T these values were 86.21, 9.31, and 36.59 μg/g, respectively (**Table 1**). The contents of TF, TP, procyanidin B2, and procyanidin B3 in CL150T was significantly higher than that in CL150CK. In contrast, the content of procyanidin B1 in CL150T was lower than that in CL150CK.

**Table 1:** Flavonoid content of different treatments.

Sample	TF (mg/g)	TP (mg/g)	Procyanidin B1 (μg/g)	Procyanidin B2 (μg/g)	Procyanidin B3 (μg/g)
CL150CK	37.31 ± 1.81 c	76.28 ± 13.85 b	212.13 ± 7.54 a	5.18 ± 2.27 bc	83.56 ± 4.64 b
CL150T	54.51 ± 2.29 a	127.77 ± 6.11 a	204.49 ± 14.89 a	54.68 ± 3.67 a	107.22 ± 5.19 a
CL102CK	37.99 ± 0.52 c	81.55 ± 5.97 b	53.86 ± 3.9 c	1.15 ± 0.31 c	12.82 ± 0.57 d
CL102T	47.54 ± 1.09 b	116.49 ± 8.11 a	86.21 ± 5.68 b	9.31 ± 1.57 b	36.59 ± 3.02 c

All data correspond to the mean values ± SD of three biological replicates. Values with different letters within the same column are significantly different (P < 0.05).

### Isolation and sequence analysis of the *CoDFR* from resistant *C. oleifera*

**Figure 2:**
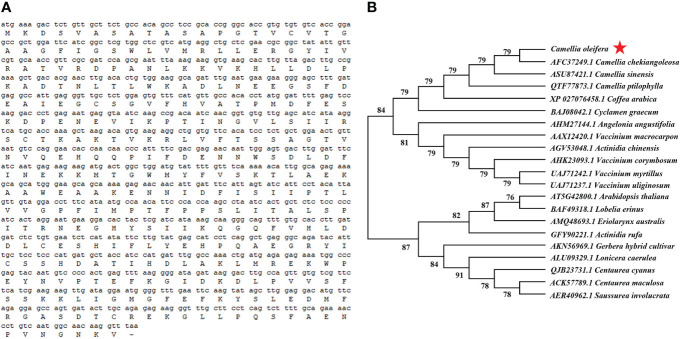
**(A)** The CDS and amino acid sequences of *CoDFR*. **(B)** The phylogenetic tree analysis of CoDFR.

The full-length CDS sequence of *CoDFR* was 1044 bp encoding 347 amino acids (**Figure 2A**). To further explore the evolutionary relationships of *CoDFR*, a phylogenetic tree was constructed using DFR amino acid sequences from different plant species. The results revealed that the CoDFR sequence has the highest homology (~79%) with DFR in *C. chekiangoleosa* (**Figure 2B**).

### Tissue-specific expression patterns of *CoDFR* gene in resistant *C. oleifera*

**Figure 3:**
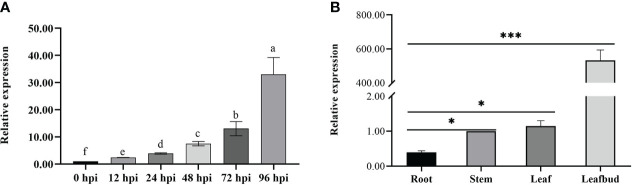
**(A)** Relative expression analysis of *CoDF*R at different time points during *C fructicola* infection **(B)** Tissue-specific expression analysis of *CoDFR* in disease-resistant line. Error bars represent ± SD from three biological repeats. "*" indicates significant difference from WT (p<0.05), "***" indicates significant difference from WT (p<0.005).

We observed that the expression of *CoDFR* gradually increased from 0 to 96 hpi, and was significantly higher than that in control (0 hpi) (**Figure 3A**). Meanwhile, we studied *CoDFR* expression in different *C. oleifera* tissues (**Figure 3B**) and found that the expression levels of *CoDFR* in leaf bud were markedly higher than that in leaf, stem, and root by more than 300-fold.

### Subcellular localization of *CoDFR*

**Figure 4:**
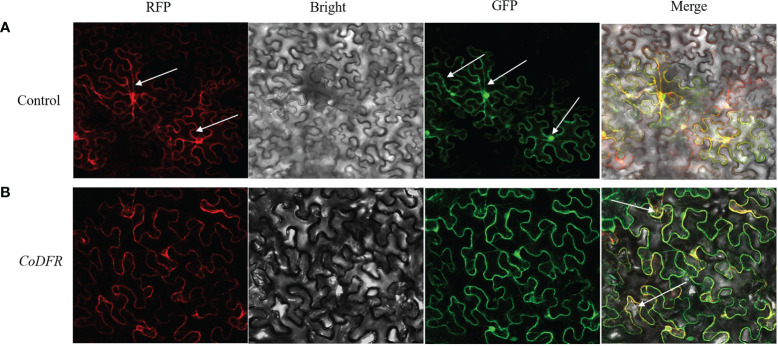
Subcellular localization of CoDFR. **(A)** Control, EV-GFP, and endoplasmic reticulum marker (35S: EV-GFP). **(B)** CoDFR, 35S: CoDFR-GFP and endoplasmic reticulum maker fusion proteins (35S: CoDFR-GFP). The white arrow shows the location of the description.

The CoDFR protein localization to the endoplasmic reticulum (ER) was predicted online by the subcellular localization prediction website (http://www.csbio.sjtu.edu.cn/bioinf/Cell-PLoc-2/). To determine the subcellular localization of CoDFR, the suspension of *A. tumefaciens* cells harboring CoDFR:: GFP (greenfluorescent protein) fusion construct was injected into tobacco leaves. As shown in **Figure 4A**, the RFP (red fluorescent protein of mCherry) signals of control was distributed in the ER and cell membrane, free-GFP signals appeared in the nucleus, cell membrane and cytoplasm, and the signals were scattered throughout the whole cell. Meanwhile, whereas the ER marker and CoDFR-GFP colocalized in ER and cell membrane (**Figure 4B**), suggesting that the *CoDFR* performs its function in the ER.

### Identification of overexpressing tobacco lines

**Figure 5:**
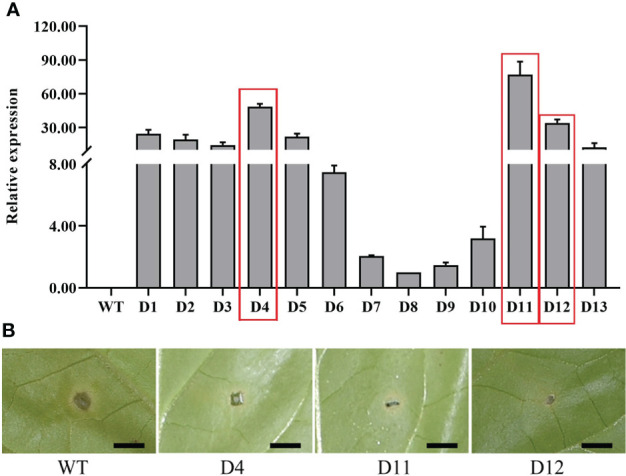
**(A)** The relative expression level of *CoDFR* in transgenic tobacco plants, red boxes indicate the three transgenic tobacco lines with high relative expression. **(B)** Phenotypes of transgenic tobacco after *Co. fructicola* infection, scale bar is 0.5 cm.

To unravel the function of *CoDFR*, we generated the transgenic tobacco lines by heterologous expression of *CoDFR*. Thirteen independent transgenic tobacco lines were screened on a hygromycin selection medium, and the relative expression levels of *CoDFR* in all the lines were determined by qRT-PCR analysis. Based on the results, we selected three transgenic lines (lines D4, D11, and D12) with higher expression of *CoDFR* for subsequent analysis (**Figure 5A**). To assess whether the transgenic tobacco had higher disease resistance, we inoculated five-week-old tobacco leaves with *C. fructicola*. Interestingly, minor necrotic lesions were observed on the transgenic leaves, in contrast to severe disease symptoms developed on wild-type (WT) leaves at 4 days post-inoculation (**Figure 5B**).

### Overexpression of *CoDFR* in tobacco enhanced the resistance to *C. fructicola* infection

**Figure 6:**
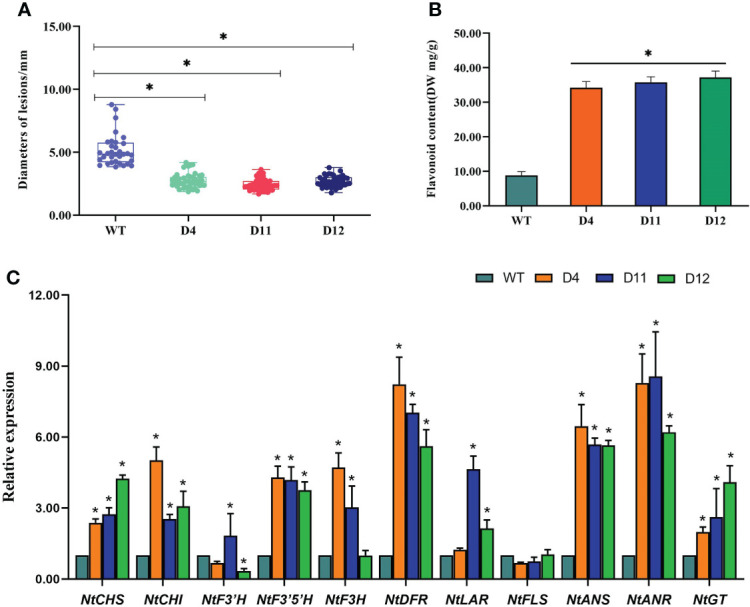
**(A)** Statistical analysis of the disease spot size after*C fructicola* infection. **(B)** The flavonoid content in transgenic tobacco. **(C)** The expression level of key genes involved in the flavonoid biosynthesis pathways. Asterisks indicate statistically significant differences from the WT (“*”*P* < 0.05).

The disease spots on transgenic tobacco were significantly smaller than those on WT (**Figure 6A**). To further investigate whether the overexpression of CoDFR has contributed to the accumulation of flavonoids, we first estimated the flavonoid content in transgenic tobacco leaves. The flavonoid content in the transgenic D4, D11, and D12 lines was found to be significantly higher than the control (WT), with a 3.89, 4.07, and 4.24-fold increase, respectively (**Figure 6B**). Furthermore, we evaluated the expression of flavonoid biosynthesis pathway genes in transgenic tobacco leaves infected with *C. fructicola* at 96 hpi. The results showed that the expression of all genes involved in the flavonoid pathway was altered in CoDFR-overexpressed tobacco leaves. *NtCHS* was up-regulated 2.38-, 2.74-, and 4.24-fold; *NtCHI* was up-regulated 5.01-, 2.54-, and 3.08- fold in D4, D11, and D12, respectively. The expression fold change of *NtF3’H, NtF3’5’H, NtF3H, NtDFR, NtLAR*, and *NtFLS* ranged from 0.34 to 1.83, 3.76 to 4.29, 1.00 to 4.71, 5.61 to 8.26, 1.24 to 4.65, and 0.68 to 1.04, respectively. *NtANS, NtANR*, and *NtGT* expression increased by 5.65~6.47, 6.21~8.56, and 1.99~4.09 fold, respectively (**Figure 6C**).

### Overexpression of CoDFR enhanced the accumulation of flavonoids in tobacco

**Figure 7:**
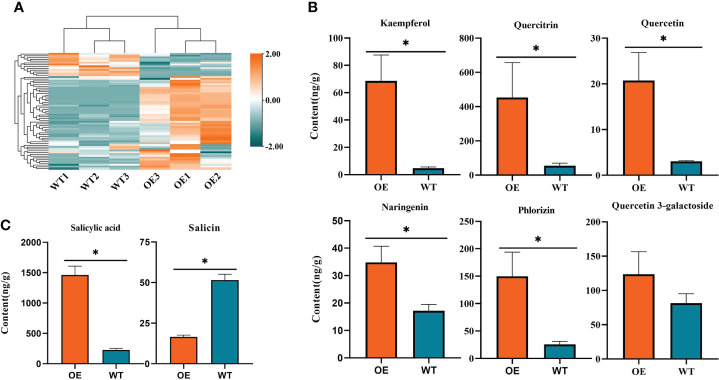
**(A)** Heatmap visualization of 65 flavonoids. The content of each flavonoid was normalized by row. **(B)** The contents of different flavonoids in tobacco expressing *CoDFR*. **(C)** The salicylic acid content in tobacco expressing *CoDFR*. “*” indicate statistically significant differences (*P* < 0.05) from the WT.

To explore the variation in the accumulation of flavonoid substances in overexpression lines, we used UPLC-ESI-MS/MS for the absolute quantification of over 130 flavonoids. A total of 65 flavonoids were identified in both wild-type and CoDFR-overexpression lines (equal mix of D4, D11, and D12 lines), including 31 up-regulated, 12 down-regulated, and 22 unchanged (A difference multiple of ≧2 or ≦0.5 was used as a screening criterion. Details are given in **Table S3**). In addition, the accumulation of 46 flavonoid derivatives was higher in the CoDFR-overexpression lines (**Figure 7A**; **Table S2**). The levels of major flavonoids such as kaempferol, quercitrin, quercetin, naringenin, phlorizin, and quercetin-3-galactoside were simultaneously increased by 14.82-, 8.24-, 6.82-, 2.03-, 5.83-, and 1.52-fold compared to control (**Figure 7B**; **Table S3**). Interestingly, the overexpression lines had higher salicylic acid but lower salicin content than the WT lines (**Figure 7C**).

## Discussion

Numerous studies have shown that plants alter their physiological state in response to adverse conditions and stress (Ali et al., 2020; Khalil et al., 2022; Velho et al., 2022). Malondialdehyde (MDA) can be used as an indicator to evaluate the extent of plasma membrane damage and the ability of plants to tolerate stress conditions (Morales and Munné-Bosch, 2019; Saadati et al., 2020; Zhang et al., 2021). In this study, MDA content in the leaves of resistant and susceptible lines was increased simultaneously after inoculation with *C. gloeosporioides*, though the overall content was less in the resistant lines compared to the susceptible plants. Therefore, it can be speculated that the MDA content is negatively correlated with disease resistance. Also, these findings suggest that the disease-resistant line suffered less damage, probably due to the disease-resistant line having a stronger scavenging system.

Pathogen infestation in plants causes alteration in many metabolic pathways and physiological responses. CAT, POD, and SOD activities are common indicators for assessing plant resistance to diseases (Faulkner and Robatzek, 2012; Prabhukarthikeyan et al., 2018). In the SOD-POD system, SOD first degrades ${\text{O}}_{2}^{-1}$ into O_2_ and H_2_O_2_, and the latter is then degraded by POD into H_2_O and O_2_ (Boscolo et al., 2003). CAT is a key enzyme in the scavenging of H_2_O_2_ to water and molecular oxygen *via* the transfer of two electrons (Mukhopadyay et al., 2012). Our results showed that compared to the control (non-inoculated plants), CAT, POD, and SOD activities increased more after *C. gloeosporioides* infection in the disease-resistant lines than in the susceptible lines. These results are consistent with the previous studies (Li et al., 2018; Pandey et al., 2021). There are two potential possibilities for disease-resistant lines to have higher conserved enzyme activities than disease-susceptible lines. The first possibility could be that during *C. gloeosporioides* infestation, substantial reactive oxygen ions are generated. Moreover, excessive accumulation of reactive oxygen species (ROS) may hamper physiological processes in plants (Wang et al., 2018; Zhang et al., 2020). To avoid cell damage due to reactive oxygen, SOD and POD form the first line of antioxidant defense against ROS (Apel and Hirt, 2004; Shi et al., 2019). The second possibility might be that POD induces lignin synthesis, which strengthens the cell wall to deter pathogenic bacteria from invading (Ma and Berkowitz, 2007; Tian et al., 2019). At the same time, H_2_O_2_ produced by SOD can either induce programmed cell death (PCD) or work as signal molecules to mediate the crosstalk within many different signaling molecules, thereby regulating the expression of genes involved in defense responses (Habibi, 2014; Zhang et al., 2014; Kaurilind et al., 2015; Niu and Liao, 2016). It has been shown that PCD is a highly regulated feature of plant immune response (Cai et al., 2020). Our study revealed that the activities of CAT, SOD, and POD showed a consistent trend of increasing and then decreasing. The probable reason is that as the duration of stress increases, the necrotic area of the spot increases, leading to a reduction in the enzyme activity per unit mass of the leaf (Yang et al., 2022). It is also possible that *C. gloeosporioides* is predominantly a biotrophic-fungi in the early stages and a necrotrophic-fungi in the later stages (da Silva et al., 2020). At the same time, POD activity was observed earlier than the peak activity of CAT and SOD, indicating that H_2_O_2_ plays an important role in disease resistance during the early infection stage in oil tea.

Plants are constantly attacked by various organisms during their life cycle, including viruses, bacteria, fungi, and others (Xin et al., 2020). Plants produce a wide range of secondary metabolites such as flavonoids, terpenoids, alkaloids, and phytohormones that initiate and/or mediate a powerful defense mechanism (Dixon, 2003; Jones and Dangl, 2006). Flavonoids play a key role in plant responses to biotic stresses (Jiang et al., 2021; Li et al., 2021). In this study, we observed that the accumulation of flavonoids in oil tea leaves enhanced following *C. gloeosporioides* infection. Interestingly, the increase in flavonoid content was higher in the resistant lines than in the susceptible lines. In a previous study, we observed a considerable accumulation of *CoDFR* transcripts in response to *C. gloeosporioides* infection (Yang et al., 2022). In the current study, we found that the expression of *CoDFR* increased with the subsequent inoculation time points. It has been shown that plants adapt to the environment through the MIR156-SPLS-DFR pathway by achieving stress tolerance (Cui et al., 2014). To substantiate the function of *CoDFR*, the specific site of expression in the cell was determined. *CoDFR* was found to be localized in the endoplasmic reticulum, which is consistent with the results described in previous studies (Singh et al., 2009). However, we also observed some fluorescence in the cell membrane, possibly because the endoplasmic reticulum is an organelle with a reticular structure inside the cytoplasm, consisting of a membrane. Therefore, the GFP fluorescence observed in the membrane could be attached to the endoplasmic reticulum (Voeltz et al., 2002). Another possibility is that CoDFR an extremely likely dual subcellular localization (Jiang et al., 2020). To further explore the function of *CoDFR*, we generated transgenic tobacco plants overexpressing *CoDFR*. The transgenic plants exhibited increased resistance to *C. fructicola*. Next, we sought to determine whether the increased disease resistance in transgenic tobacco is related to the amount of flavonoid accumulation. In the current study, the expression of flavonoid synthesis pathway genes such as *NtCHS, NtCHI, NtF3’5’H, NtDFR, NtANS, NtANR*, and *NtGT* was found to be increased to different degrees in transgenic tobacco. The high expression of genes related to the flavonoid synthesis pathway in transgenic tobacco may be due to the promotion of secondary metabolite synthesis through a feedback regulatory mechanism. At the same time, it provides precursors and promotes the synthesis of downstream products (Naing et al., 2018; Zhao et al., 2020; Liu et al., 2022b). It has been shown that overexpression of DFR genes results in an increased accumulation of flavonoids, further enhancing disease resistance in the transgenic plant (Kumar et al., 2013; Kim et al., 2017; Gu et al., 2018). We observed that three transgenic lines overexpressing *CoDFR* showed 3.89, 4.07, and 4.24-fold increases in flavonoid content. It is speculated that the higher expression of flavonoid synthesis-related genes in overexpressing tobacco may leads to increased flavonoid content thus resulting in greater resistance to anthracnose in overexpressing lines.

Flavonoids are numerous, and the function of most flavonoids in response to biotic stresses has been reported, which varies in response to pathogens (Ekuadzi et al., 2014; Murata et al., 2020; Su et al., 2021). We detected 43 differentially accumulated flavonoids between the overexpression and wild-type lines in the current study. Most flavonoids in transgenic tobacco accumulate in larger amounts, especially eight of the flavonoid pathways (ko00941) components. For example, the kaempferol content in the tobacco overexpressing *CoDFR* was 14.82-fold higher than in the wild-type. Kaempferol, a flavonoid, is a precursor for quercetin and myricetin synthesis, and its native and downstream products have certain antibacterial effects (Ma et al., 2017). The accumulation of quercitrin and quercetin in overexpressed tobacco was 8.24- and 6.82-fold higher compared to wild-type tobacco. Quercitrin is a flavonoid with antioxidant and antimicrobial activity, and it can also induce downstream signaling (Li et al., 2016; Xing et al., 2017; Hardiyanti et al., 2019). More interestingly, the salicylic acid (SA) content in the overexpression lines was 6.43-fold higher than that in the WT, while the salicin content was only 3-fold lower in transgenic tobacco as compared to the WT. Hydrolysis of salicin produces 2-Hydroxybenzyl alcohol, which is readily oxidized to produce salicylic acid. This may result in high salicylic acid content and low salicin content (Vlachojannis et al., 2011). Numerous studies have shown that SA is a defense-related phytohormone that plays a key role in plant resistance to different microbial pathogens, such as viruses, bacteria, and fungi. (Zhang and Li, 2019; Koo et al., 2020). Salicylic acid enhances the defense of poplar (*Populus nigra* L.) against fungal diseases by promoting the accumulation of catechins and procyanidins (Ullah et al., 2019; Yoosomboon et al., 2021). In our study, the SA content and *NtDFR* expression of overexpressed tobacco lines were significantly higher than those of WT lines. However, in our results, the direct downstream products of *DFR*, such as leucodelphinidin, were not detected. Also, the contents of secondary downstream products, such as epicatechin and gallocatechin, were increased, though the difference was less than 2-fold. We speculate that this result may be due to the substrate specificity of different DFR enzymes (Johnson et al., 1999; Ruan et al., 2022).

## Conclusion

In conclusion, the current study revealed the physiological status of *C. oleifera* leaves was changed, and flavonoid content was increased in the leaves after *C. gloeosporioides* infection. Further, we functionally characterized *CoDFR* from the resistant *C. oleifera* variety following *C. gloeosporioides* inoculation. The expression of *CoDFR* was positively correlated with the time course of *C. gloeosporioides* infestation. Overexpression of *CoDFR* in *Nicotiana tabacum* L. increased salicylic acid content and modulated the expression of genes involved in flavonoid pathways, which promoted the accumulation of flavonoids and thereby increased resistance to anthracnose. Collectively, these findings may be relevant for increasing the resistance of oil-tea to fungal pathogens. Furthermore, it provided the implications of our findings for the broader resistance of plants to necrotrophic pathogens.

## Data availability statement

The original contributions presented in the study are included in the article/**Supplementary Material**. Further inquiries can be directed to the corresponding authors.

## Author contributions

XY and RZ conceived this project. CY, PW, and XY designed experiments and interpreted the results. CY wrote the manuscript. YC, BY, LL, and JC performed the experiments and analyzed the data. XY provided experimental materials and funds. All authors contributed to the article and approved the submitted version.

## Funding

This research was financially supported by National Key R&D Program of China (2019YFD1001602).

## Conflict of interest

The authors declare that the research was conducted in the absence of any commercial or financial relationships that could be construed as a potential conflict of interest.

## Publisher’s note

All claims expressed in this article are solely those of the authors and do not necessarily represent those of their affiliated organizations, or those of the publisher, the editors and the reviewers. Any product that may be evaluated in this article, or claim that may be made by its manufacturer, is not guaranteed or endorsed by the publisher.
